# Supplementary material for: Conditional chemoconnectomics (cCCTomics) as a strategy for efficient and conditional targeting of chemical transmission
Source: eLife. 2024 Apr 30;12:RP91927. doi: 10.7554/eLife.91927 (PMC11060718; doi:10.7554/eLife.91927)
Supplement: Figure 3—source data 3. [file elife-91927-fig3-data3.zip › Figure 3 source data3 part 1.zip!/Figure 3-Source data 3-part1/1082-9-CRISPResso_Report_zod2ewbs.zip!/CRISPResso_on_1082_11/4c.Quantification_window_insertion_deletion_substitution_locations.pdf]

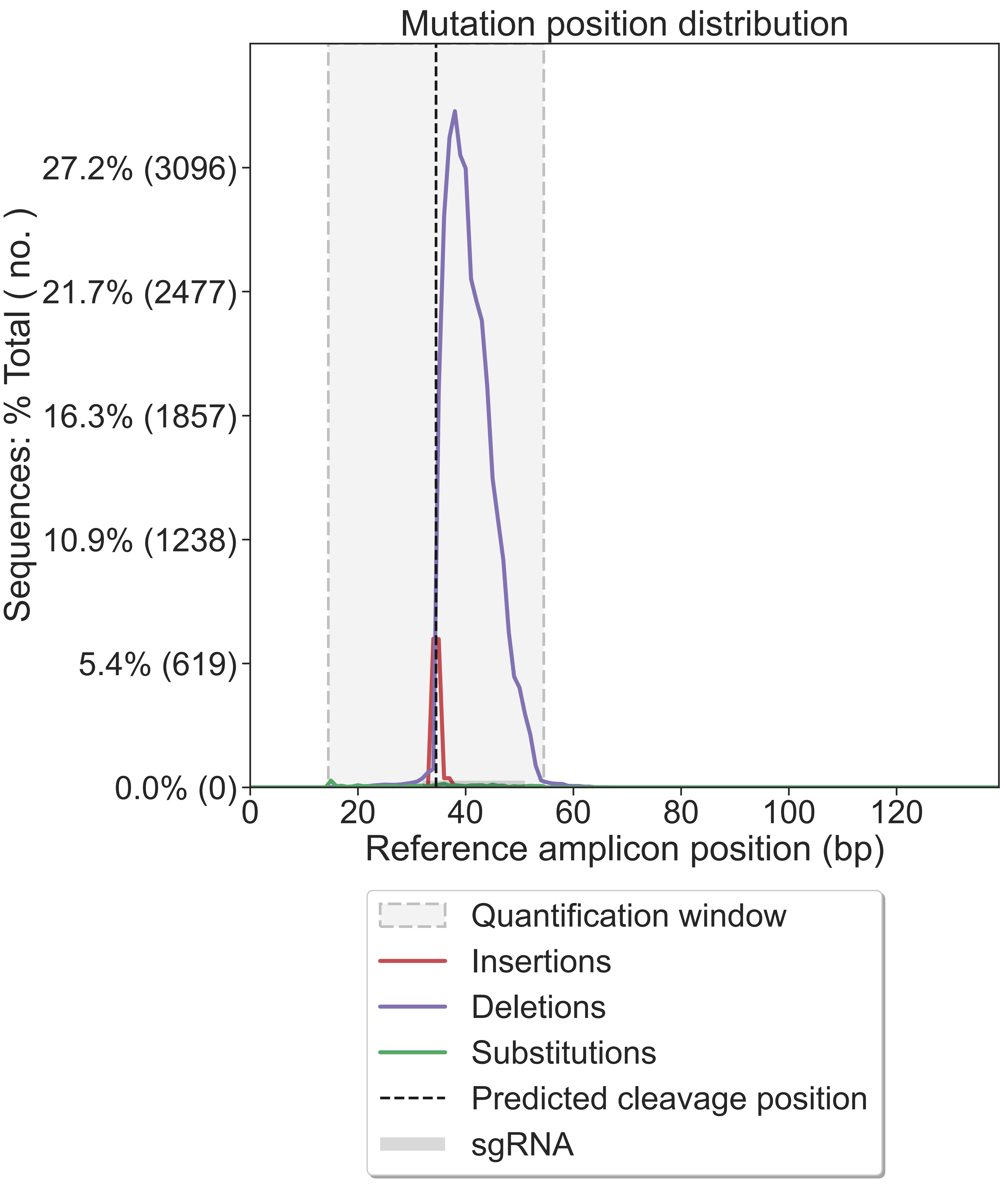

Mutation position distribution
Sequences: % Total (no.)
27.2% (3096)
21.7% (2477)
16.3% (1857)
10.9% (1238)
5.4% (619)
0.0% (0)
0
20
40
60
80
100
120
Reference amplicon position (bp)
Quantification window
Insertions
Deletions
Substitutions
Predicted cleavage position
sgRNA
